# Application of TOPSIS algorithm in describing bacterial cellulose-based composite hydrogel performance in incorporating methylene blue as a model drug

**DOI:** 10.1038/s41598-023-29865-6

**Published:** 2023-02-16

**Authors:** Touraj Amrabadi, Elham Jalilnejad, Seyed Mohammad Amin Ojagh, Farzaneh Vahabzadeh

**Affiliations:** 1 Department of Chemical Engineering, Amirkabir University of Technology (Tehran Polytechnic), Tehran, Iran; 2 Department of Chemical Engineering, Urmia University of Technology, Urmia, West Azerbaijan Iran; 3 Department of Chemistry, McGill University, Montreal, QC Canada

**Keywords:** Biomaterials, Drug delivery, Pharmaceutics, Biomedical engineering, Chemical engineering, Chemical engineering, Materials chemistry, Polymer chemistry

## Abstract

A multi-component hydrogel was developed using bacterial cellulose, alginate, and gelatin with the aid of glycerol as trihydric alcohol which participates in re-distribution of hydrogen bonds in the test system. FTIR, XRD, SEM, and TGA as instrumental techniques were used to structurally characterize the physical/chemical properties of the formed composite hydrogel. By using an exponential equation, swelling behavior of the hydrogel was evaluated. By incorporating a model drug (methylene blue—MB) in the formed hydrogel, experiments were directed to study release characteristics of the MB where the medium solution for the release was prepared at four different pHs. The maximum cumulative drug release at pH 2.8, 6, 7.4, and 9 were 42.8, 63, 80, and 84.5%, respectively. Data fitting process was carried out using five kinetic models (Korsmeyer-Peppas, Higuchi, Hopfenberg, zero-order, and first-order equations) and the preferred kinetic model at each pH was estimated by applying TOPSIS algorithmic technique. The adsorption capacity of the hydrogel in relation to MB was determined while thermodynamic properties of this relationship were quantified ($\Delta{\text{H}}_{\text{ad}}^{0}= \text{ } -\text{99.95 kJ} \, {\text{mo}}{\text{l}}^{-{1}}$ and $\Delta{\text{S}}_{\text{ad}}^{0}= -\text{0.237 kJ} \, {\text{mo}}{\text{l}}^{-{1}} {\text{K}}^{-{1}}$). The results of the present study were in favor of the potential usage of the developed composite hydrogel in drug delivery systems.

## Introduction

Attentions in medical fields were directed in recent years toward using biopolymer-based composite hydrogels mainly because of their biocompatibility, biodegradability, and non-toxicity and bacterial cellulose (BC) is the most popular one among natural polymers^1^.

A recent study on the kinetics of cellulose synthase catalyzed cellulose synthesis in *Gluconacetobacter hansenii* has shown the involved process in detail, where initiation, elongation, and termination (release of the synthesized cellulose from enzyme) are three phases through which structural orderliness increases gradually and step-by-step^2^: Parallel alignment of the formed chains of interconnected D-glucose units through glycosidic covalent bonds is lengthwise while hydrogen bonds (inter/intra-linkages) and Van der Waals forces ultimately give intertwined structures assignable to native cellulose as cellulose I microfibril (a regular pattern of long and entangled chains of the formed cellulose I can not be maintained throughout the structure and paracrystalline is the result of this structural consideration)^3^. Several microfibrils are held together extensively, and the ribbons structures are then formed and stacked on each other and placed outside of the bacterial cell^3^. The complexity of these types of structures is high, and they play a role as cell protective coverage, which are categorized as microbial biofilms^3^. The aforementioned layer gives the ability to the producer bacteria to cope better with environmental stresses where damages may be caused by biotic and abiotic factors.

Alginate (Alg) is a natural polysaccharide extracted from algae, and β-d-mannuronic acid and α-l-guluronic acid are constituents of this polymer where these uronic acids could extensively participate in the cross-linking process in the presence of polyvalent metal cations. The alginate polymer eventually reaches the breakup point of its “egg-box” structure, focusing on calcium ions as the preferred ones and replacing them with sodium ions on the carboxylate groups. This type of experiment (calcium ion diffusion through the network monitored by the electron paramagnetic resonance method) is likely to result in increasing distances between the polymer chains^4^.

Many studies in recent years have been directed toward the production of gelatin-based hydrogels^5–8^. Attention to this subject first is toward the highly interconnected triple helix in collagen in terms of the unique amino acid sequences which have made this protein easily affected by environmental factors^9^. Production of gelatin, in fact, is a result of heat treatment and partial hydrolysis of collagen as a fibrous protein in which the polypeptide chains are no longer in regular association with each other (i.e., gelatin as a denatured product of collagen)^9^.

Combining BC, Alg, and Gl, physical linkages were formed between suitable chemical chains utilizing calcium ions and with the aid of glycerol as trihydric alcohol capable of participating in the formation of hydrogen bonds through its hydroxyl groups and other functional groups present in the polymer chains (–OH, –NH, –COO^−^, –CO). Cross-linking between these linked polymers led to the formation of BC-based composite hydrogel (BC-based CH). The experiments in the present study were first directed toward using FTIR, XRD, TGA, and SEM analyses, and the formed composite hydrogel thus was structurally characterized.

Further points of interest were to quantitatively evaluate the behavioral functionality of the composite hydrogel in terms of its swelling capacity and drug adsorption using Langmuir and Freundlich isotherms. Extent of the relevant processes was thermodynamically quantified.

Drug release from composite hydrogel involves several steps which occur sequentially/ simultaneously, starting from water absorption, diffusion, and drug transport through the polymeric network. Physical entanglement/disentanglement of the hydrated polymer, swelling, drug dissolution and diffusion into the test solution all indicate that several mechanisms are involved in drug release^10,11^. With use of methylene blue (MB) as a model drug, release kinetic was described mathematically in the present work. Five different models were used namely Korsmeyer-Peppas, Higuchi, Hopfenberg, zero-order, and first-order equations. The quality of a model is explainable by its predictive ability as related to its accuracy in the prediction. Thus, the potentiality of TOPSIS application as a multiple-criteria decision-making (MCDM) method in the present study was evaluated.

## Materials and methods

### Materials

BC membranes were purchased from Nano Novin Polymer Company in Iran (${20} \, \times \, \text{30 } \times \, \text{0.4}$ cm) and were treated using 0.1 M NaOH solution (90 °C for one hour), and then were washed with distilled water (DW) until the pH of solution reached neutral pH ~ 7. Gl (110 bloom), Alg (extracted from brown algae), and calcium chloride were all purchased from local markets provided as analytical grade (Sigma-Aldrich and Applichem GmbH).

### Preparation of the BC-based CH

The treated BC membranes were divided into 10 mm by 10 mm by 4 mm pieces, and 200 ml of DW was added. The mixture was then transferred into a homogenizer equipped with the tap water circulating system to maintain the condition at moderate temperature (16,000 rpm for 15 min, HO4 Edmund Bühler 7400 Tübingen, Germany). The concentration of prepared BC slurry was adjusted to 2 wt%. This was done via centrifugation (10,000 rpm for 10 min) and the supernatant was removed (Biofuge Stratos, Thermo Scientific, USA). With regard to findings reported by Chiaoprakobkij et al., preliminary work was performed based on one-at-a-time experiment, and the results were informative to prepare 10 g of the composite hydrogel^12^. The procedure below was followed: Gl powder (0.3 g) was first mixed with a proportional amount of DW for 1 h using a magnetic heater stirrer (500 rpm and 60 °C). Relevant portions of Alg (0.05 g), glycerol (0.1 g), and NaCl (0.1 g) were added and stirring was continued until a homogenous solution was obtained. Then, 5 g of BC slurry was added, and the mixture was stirred for 1 h at 500 rpm. The homogenous mixtures were placed onto sterile petri plates, and the plates were dried for 72 h at about 35 °C in a vacuum oven (H. Jürgens & Co., Bremen, Germany). Each of the dried samples was mixed with 50 ml of ${\text{CaC}}{\text{l}}_{2}$ solution (2.5% w/v) using shaker incubator (Kühner shaker, Switzerland, 70 rpm for 60 min). Thereafter, the cross-linked composite hydrogels were rinsed with DW to remove any unreacted agents. The formed composite hydrogels were cut into $\text{1 } \,\times\, \text{1 \, cm}$ pieces, followed by drying at 45 °C for 4 h.

### Instrumental analyses

Fourier transform infrared spectra were recorded with the spectrometer (Thermo Fisher Scientific Co. Ltd., MA, USA). The data were collected from 4000 to 600 ${\text{c}}{\text{m}}^{-{1}}$ in the transmission mode.

The orderly arrangements of the atoms-molecules in a material give specified patterns to the sample, and this is the basis of material crystallinity, where the responses to exposure to X-rays are measured in XRD spectroscopy. The intensity and scattering angle of the X-rays that leave the sample then is recorded (by specifying the range from $\text{2}$θ such as 5°–40°). By following the instruction manual, measurements were made with the use of Ni filter at room temperature with the voltage and current generated at 40 kV and 40 mA, respectively (CuKα as a radiation source, KEFA XRD, Panalytical Inc., Netherlands).

Peaks in XRD diffractograms were detected visually and the results were analyzed by Origin Pro software (9.8 version). The relevant plot then was used for crystallinity calculation according to the expression given below:

1 $$crystallinity\, index=\frac{{I}_{c}}{{I}_{c}+{I}_{a}}\times 100$$

where ${\text{I}}_{\text{c}}$ represents the area of the total crystalline phase and ${\text{I}}_{\text{a}}$ indicate the area of the amorphous phase.

Using field emission-scanning electron microscopy (Tescan ‘Mira 3’, Czech Republic), FE-SEM studies of the samples were performed. The non-conducting behavior of cellulose is a possible cause of charge buildup on the sample surface, and this can affect quality of developing images unfavorably, and this can be prevented by gold plating the surface. The details of the procedure are given elsewhere^13^.

Measurements were made using a thermal analyzer to assess the thermal stability of the prepared BC and BC-based CH (Mettler Company, USA). By weighing a 5 mg sample and placing it in an aluminum pan, the heating process was done with the following specification: heating rate of 10 °C/min under ${\text{N}}_{2}$ atmosphere with a flow rate of 50 ml/min from 25 to 500 °C. By obtaining the first derivative thermogravimetric curves (DTG), attempts were directed to find the temperature at which maximum weight loss would occur.

Prepared BC and BC-based CH in a liquidic medium is a type of colloidal dispersion, and its stability could be measured quantitatively by the zeta potential–the extent of the resistance to flocculation, which is an indicator of inadequate electrostatic charge at the particle surface, can be estimated (Cordouan Tech, WALLIS, France).

The specific surface area and pore volume of samples were determined by ${\text{N}}_{2}$ using autosorb-1-MP gas sorption system (Quantachrome Corporation, Austria) operated based on the BET concept.

### Swelling characteristic and MB loading experiments

The swelling behavior of BC-based CH samples at different pHs (pH 2.8–9 using “phosphate buffer”) was analyzed by the gravimetric method. In brief, the dried sample was initially weighed (${\text{W}}_{0}$) and then immersed in solutions having a specified pH at 37 °C for 45 min. The swollen samples were regularly removed from the solutions and re-weighed (${\text{W}}_{\text{s}})$ immediately after the excess media was blotted with filter paper and the swelling capacity was measured: $\text{swelling \, capacity}=({\text{W}}_{\text{s}}-{\text{W}}_{\text{d}}\text{)/}{\text{W}}_{\text{s}}$. The swelling rate was determined using the following exponential equation^14^:

2 $${S}_{t}={S}_{e}(1-{e}^{-t/r})$$

where ${\text{S}}_{\text{t}}$ represent swelling capacity at time t, ${\text{S}}_{\text{e}}$ is the equilibrium swelling where the swelling reaches a maximal value, and r as the rate parameter indicates the time by which swelling capacity reaches 0.63 of the equilibrium swelling.

In performing drug loading experiments, aqueous solutions of MB at varying concentrations from 20 to 100 mg/l were prepared using 100 ml Erlenmeyer flasks each containing 15 ml of the test solution. The amount of the BC-based composite hydrogels added to each flask was 4 mg. The flasks were incubated in a shaker incubator (70 rpm—Kühner shaker, Switzerland) at three temperatures (27 °C, 37 °C, and 47 °C) for three days. MB in the test solution was estimated spectrophotometrically (665 nm—UV–Vis Jasco, Japan).

### Adsorption isotherm and process thermodynamic

Langmuir (Eq. 3) and Freundlich (Eq. 4) equations were used to quantitatively describe the relationship between MB and BC-based CH in terms of adsorption isotherm:

3 $${Q}_{e}=\frac{{Q}_{m}{K}_{L}{C}_{e}}{1+{K}_{L}{C}_{e}}$$

4 $${Q}_{e}={K}_{F}{C}_{e}^\frac{1}{n}$$

where ${\text{Q}}_{\text{e}}$ (mg/g) is the amount of MB as the adsorbate adsorbed per unit of mass of BC-based CH as the adsorbent at equilibrium, ${\text{Q}}_{\text{m}}$(mg/g) is an indicator of chemisorption capacity (theoretically defined as monolayer adsorption), ${\text{C}}_{\text{e}}$ (mg/l) is the MB concentration in the bulk solution at equilibrium, and ${\text{K}}_{\text{L}}$ (l/mg) is Langmuir constant. The value of ${\text{Q}}_{\text{e}}$ can be determined with the use of the following equation:

5 $${Q}_{e}=\frac{V\left({C}_{0}-{C}_{e}\right)}{W}$$

where V is the total volume (l), W is the quantity of BC-based CH (g), and ${\text{C}}_{0}$ (mg/l) is the initial concentration of MB.

Further note is to determine ${\text{R}}_{\text{L}}$ constant which equals to ${1}\text{/(}{1} \, {+} \, {\text{K}}_{\text{L}}{{\text{C}}}_{0}\text{)}$. The value of this dimensionless constant indicates the tendency of adsorption isotherm to follow the following pattern: ${\text{R}}_{\text{L}}$ > 1 as an unfavorable trend of event, ${\text{R}}_{\text{L}}$ = 1 as a linear pattern, ${0} \, {<} \, {\text{R}}_{\text{L}} \, {<} \, {1}$ as a favorable trend, and ${\text{R}}_{\text{L}} \, {=} \, {0}$ as an irreversible event.

Further note was to consider ${\text{K}}_{\text{F}}$ as the Freundlich constant, which is indicative of relative adsorption capacity of the BC-based CH and 1/n is the measure of the intensity of adsorption, and the higher the 1/n value, the more favorable would be the adsorption.

The usual practice in estimating free energy change is to use van’t Hoff equation in which the equilibrium constant (${\text{K}}_{\text{eq}}$) is definable in terms of enthalpy and entropy change:

6 $$\Delta {G}^{0} = -RT\mathrm{ln}{K}_{eq}$$

7 $$\Delta {G}^{0}=\Delta {H}^{0}-T\Delta {S}^{0}$$

8 $$\mathrm{ln}{K}_{eq}=\frac{\Delta {S}^{0}}{R}-\frac{\Delta {H}^{0}}{R}(\frac{1}{T})$$

where R is the universal gas constant (8.314 J/mol K) and T is the temperature (K). By plotting ${\text{ln}}{\text{K}}_{\text{eq}}$ vs 1/T, the quantities of enthalpy and entropy would be determined.

### Drug release experiments

To assess the cumulative release of the model drug quantitatively, the dried MB-loaded BC-based CHs were submerged in 100 mL of the prepared solutions (pH 2.8–9) incubated in a shaker incubator at 70 rpm and 37 °C for 72 h. Appropriate aliquots were taken out of the test solutions at predefined intervals and replaced with an equal amount of fresh medium. The concentration of released MB was spectrophotometrically determined at 665 nm. The expression of the amount of MB release at time ‘t’ divided by the total amount of MB loaded into BC-based CH was used to show the percentage of the cumulative release of MB from the system.

Quantitatively obtained drug release data were used to evaluate release kinetics via the application of Korsmeyer-Peppas, Higuchi, Hopfenberg, zero-order, and first-order models (Eqs. 9–13):

9 $$\frac{{M}_{t}}{{M}_{\infty }}={k}_{KP}{t}^{n}$$

10 $$\frac{{M}_{t}}{{M}_{\infty }}={k}_{h}{t}^\frac{1}{2}$$

11 $$\frac{{M}_{t}}{{M}_{\infty }}=1-{\left[1-\frac{{k}_{0}^{{\prime}}t}{{C}_{0}a}\right]}^{m}$$

12 $$\frac{{M}_{t}}{{M}_{\infty }}={k}_{0}t$$

13 $$\frac{{M}_{t}}{{M}_{\infty }}=1-{e}^{-{k}_{1}t}$$

where ${\text{M}}_{\text{t}}$ is the cumulative amount of MB released, and M_∞_ is the cumulative amount of MB released at infinite time, ${\text{k}}_{0}$ and ${\text{k}}_{1}$ are the zero-order and first-order rate constant, respectively. ${\text{k}}_{\text{h}}$ is the Higuchi dissolution constant, ${\text{k}}_{\text{KP}}$ is the Korsmeyer–Peppas constant, and ‘n’ is the release exponent^7,15^. ${\text{k}}_{0}^{\prime}$ is the zero-order rate constant describing the degradation of polymeric network (surface erosion), ${\text{C}}_{0}$ is the initial MB drug loading to the polymeric network, ‘a’ is the network’s half thickness and ‘m’ is an exponent that varies with the geometry of the test system: m = 1, 2, and 3 for the slab, cylindrical, and spherical geometry, respectively^10^.

### Data analysis and theoretical principles

Each piece of information was collected in triplicate, shown as mean $\pm$ standard deviation (relevant error bars are shown in each figure). A non-linear approach was considered for processing data obtained in the release experiments (Origin Pro software, version 9.8). The prediction quality of the model in MB release kinetic was estimated using following expressions:

The coefficient of determination:

14 $${R}^{2}=1-\frac{{\sum }_{i=1}^{n}{{(y}_{i}-{\widehat{y}}_{i})}^{2}}{{\sum }_{i=1}^{n}{\left({y}_{i}-\overline{y }\right)}^{2}}$$

Root mean square error:

15 $$RMSE=\sqrt{\frac{1}{n}{\sum }_{i=1}^{n}{{(y}_{i}-{\widehat{y}}_{i})}^{2}}$$

Chi-square:

16 $${\upchi }^{2}={\sum }_{\mathrm{i}=1}^{\mathrm{n}}\frac{{\left({\mathrm{y}}_{\mathrm{i}}-{\widehat{\mathrm{y}}}_{\mathrm{i}}\right)}^{2}}{{\widehat{\mathrm{y}}}_{\mathrm{i}}}$$

Akaike Information Criterion^16^:

17 $$AIC=n\left(\mathit{ln}(residual \, sum \, of \, squares)\right)+2p$$

where ${\text{y}}_{\text{i}}$, ${\widehat{\text{y}}}_{\text{i}}$, and $\stackrel{\mathrm{-}}{\text{y}}$ are the experimental response for the ith observation, calculated value of ${\text{y}}_{\text{i}}$, and the average of observations, respectively. ‘n’ is the number of observations obtainable experimentally and p is the number of parameters in the model.

A brief description of steps used in using the TOPSIS technique in the present study can be considered as follows^17^:

1. The collected data in the release experiments are arranged in a mathematical matrix with 5 rows and 4 columns labeled as decision matrix (DM).
  $$\begin{array}{*{20}c} {} & {C_{1} } & {C_{2} } & \cdots & {C_{n} } \\ {A_{1} } & {x_{11} } & {x_{12} } & \cdots & {x_{1n} } \\ {A_{1} } & {x_{21} } & {x_{22} } & \cdots & {x_{2n} } \\ \ldots & \ldots & \ldots & \ldots & \ldots \\ {A_{m} } & {x_{m1} } & {x_{m2} } & {} & {x_{mn} } \\ \end{array}$$
  ${\text{A}}_{\text{i}}$ (i = 1, 2, …, m) is a symbol for alternatives and ${\text{C}}_{\text{j}}$ (j = 1, 2, …, n) is a symbol for criteria. The alternatives indicate kinetic release models described as Eqs. (9)–(13). The criteria are measurements that were used in this study to evaluate the model’s performance in terms of its prediction accuracy (Eqs. 14–17).
2. By considering ${\text{x}}_{\text{ij}}$ which represents the rating of alternative ${\text{A}}_{\text{i}}$ with respect to criterion ${\text{C}}_{\text{j}}$, the normalized decision matrix is calculated:
  18 $${\overline{X} }_{ij}=\frac{{x}_{ij}}{\sqrt{\sum_{i=1}^{m}{x}_{ij}^{2}}}$$
3. By assigning the weight to each criterion as related to an alternative, the weighted normalized decision matrix is developed:
  19 $${V}_{ij}={W}_{j}\times {\overline{X} }_{ij,}$$
  where $\sum_{\text{j=1}}^{\text{n}}{{\text{W}}}_{\text{j}}= \text{1}$ and in this study, the same weight is provided for all variables $({\text{W}}_{\text{j}}=\text{1/n}{)}.$
4. To find the best alternative in the TOPSIS method, it is to calculate the Euclidean distances of each alternative from the positive and negative ideal solutions (${\text{S}}_{\text{i}}^{+}$ and ${\text{S}}_{\text{i}}^{-}$). The following expressions show that the basis of the positive ideal solution (PIS) determination is to find the maximum value for each ${\text{V}}_{\text{ij}}$ which corresponds to a particular criterion in the criteria set for an alternative (shown as set ‘J’) and at the same time the values of this ${\text{V}}_{\text{ij}}$ are minimum for other criteria that are related to that alternative (shown as set ‘$\text{J}^{\prime}$’):
  20 $$PIS=\left\{{V}_{1}^{+}, \dots , {V}_{n}^{+}\right\}=\{\left[\mathrm{max}\left({V}_{ij}\right)if j\in J\right];\left[\mathrm{min}\left({V}_{ij}\right)if j\in J{^{\prime}}\right]\}$$
  21 $${S}_{i}^{+}=\sqrt{{\sum }_{j=1}^{n}{\left({V}_{j}^{+}-{V}_{ij}\right)}^{2}}$$
  A similar concept is considered for the negative ideal solution (NIS) determination:
  22 $$NIS=\{{V}_{1}^{-}, \dots , {V}_{n}^{-}\}=\{\left[\mathrm{min}\left({V}_{ij}\right)if j\in J\right];\left[\mathrm{max}\left({V}_{ij}\right)if j\in J{^{\prime}}\right]\}$$
  23 $${S}_{i}^{-}=\sqrt{{\sum }_{j=1}^{n}{\left({V}_{j}^{-}-{V}_{ij}\right)}^{2}}$$
5. The final step is ranking the alternatives (kinetic models) using the performance score (${\text{p}}_{\text{i}}$):
  24 $${p}_{i}=\frac{{S}_{i}^{-}}{{S}_{i}^{+}+{S}_{i}^{-}}$$
  Using the TOPSIS method, release kinetic study was separately carried out at test pHs.

## Results and discussion

### Structural characterization

**Figure 1:**
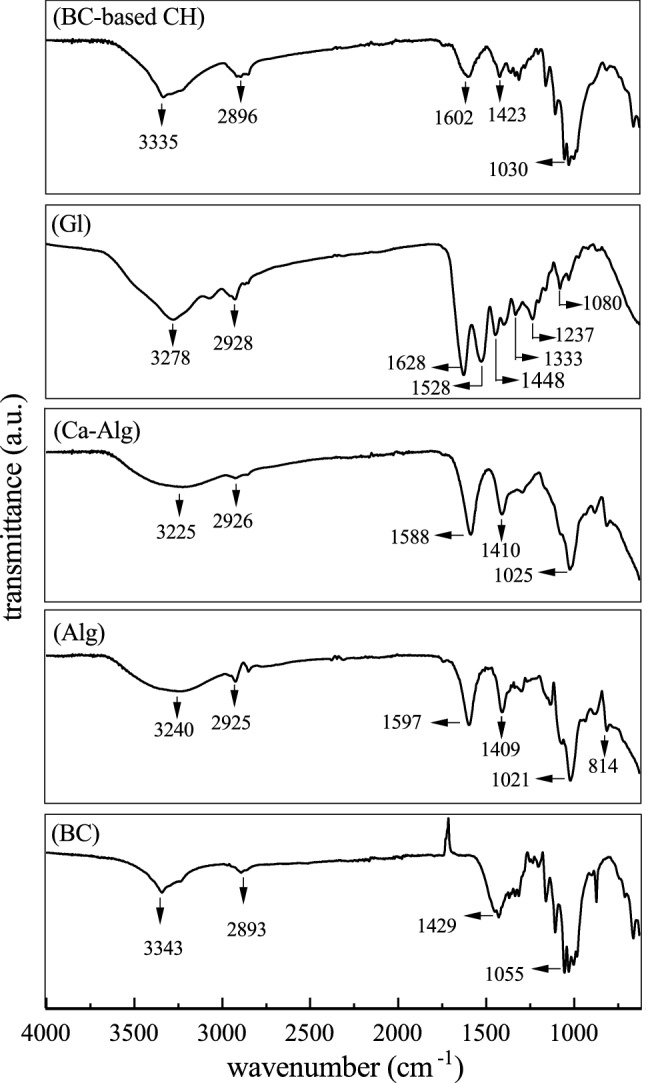
FTIR spectra of the samples of each biopolymer used in the present study before being combined to prepare the composite hydrogel (BC-based CH).

FTIR spectra indicate molecular composition of the samples in terms of the presence of functional groups and infrared spectral characteristics for the BC, Alg, Ca-Alg, Gl, and BC-based CH samples are shown in Fig. 1. Stretching and bending modes are the two simplest types of vibrational motions in an infrared-active molecule^18^. Symmetric stretch and asymmetric stretch are two types of stretching vibration, and scissoring, rocking (in plane), wagging, and twisting (out of plane) are assigned to complex types of bending vibration^18^. The spectrum of BC membrane shows, for instance, strong signals at 3343, 2893, 1429 and 1055 ${\text{c}}{\text{m}}^{-{1}}$ and these absorption peaks are attributed to O–H, –CH stretching, C–H asymmetric angular deformation, and C–OH stretching in alcohols, respectively. The findings are in agreement with those reported in the literature (Fig. 1)^12,19–22^.

FTIR characteristic bands for pure sodium alginate are related to –OH stretching (broadband around 3240 ${\text{c}}{\text{m}}^{-{1}}$), C–H asymmetric stretch (2925 ${\text{c}}{\text{m}}^{-{1}}$), asymmetric and symmetric stretching of –${\text{CO}}{\text{O}}^{-}$ group at 1597 and 1409 ${\text{c}}{\text{m}}^{-{1}}$, respectively and C–O–C stretching (1021 ${\text{c}}{\text{m}}^{-{1}}$)^19,23^. Moreover, the stretching vibration band observed at approximately 814 ${\text{c}}{\text{m}}^{-{1}}$ is specific to the mannuronic acid^12^. The characteristic peaks of Ca-Alg were similar to those of Alg, with some slight shifts, such as in the bands related to the –${\text{CO}}{\text{O}}^{-}$ stretching at 1588 and 1410 ${\text{c}}{\text{m}}^{-{1}}$, which may be in terms of the association of this group with the calcium ion (alginate cross-linked by ${\text{C}}{\text{a}}^{2+}$)^19,24^. The characteristic features of pure gelatin comprise the absorption peaks at 3278, 2928, and 1448 ${\text{c}}{\text{m}}^{-{1}}$ corresponding to N–H stretching, aliphatic C–H stretching, and C–H bending, respectively. Besides, absorption peaks at 1628, 1528, 1333 and 1237 ${\text{c}}{\text{m}}^{-{1}}$ were ascribed to the C=O stretching vibration (amide-I), N–H bending vibration (amide-II), C–N stretching vibration and N–H bending vibration, respectively^12,19,20^.

In BC-based CH, –OH stretching and asymmetric C–H stretch appeared at 3335 and 2896 ${\text{c}}{\text{m}}^{-{1}}$, respectively. The bands related to the asymmetric and symmetric stretching of –${\text{CO}}{\text{O}}^{-}$ group and C–O–C stretching shifted to 1602, 1423, and 1030 ${\text{c}}{\text{m}}^{-{1}}$, respectively^8,19^. The amino group peaks of gelatin were not clearly visible in the BC-based CH, which may be the result of the formation of complexes between anionic and cationic side chains (i.e., Alg, BC, and Gl)^12,19^.

Cellulose as a crystallin substance retains its arrangement for a long time unless its complex structure including crystallin and amorphous senses external stress. Any method for calculating crystallinity based on the two-phase model runs into several issues that have been linked to this idea. For instance, in the synthesis of cellulose and in the pathway of reaching the microfibril network, paracrystalline cellulose development is clearly sensed and its amount in cotton cellulose (33%) is found to be very close to crystalline cellulose content (31.8%)^3,25^.

**Figure 2:**
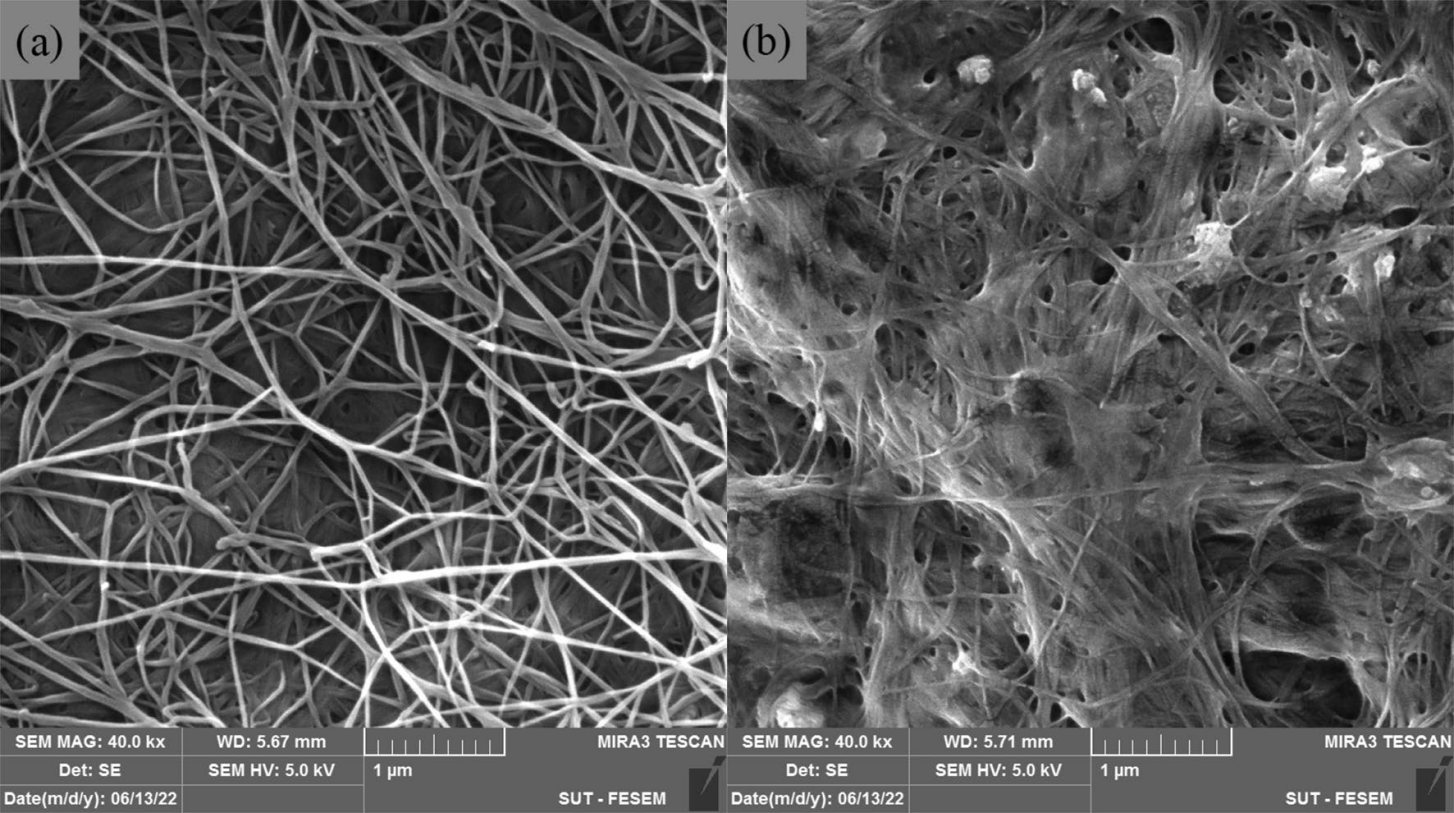
SEM images of the surfaces of (**a**) BC and (**b**) BC-based CH.

**Figure 3:**
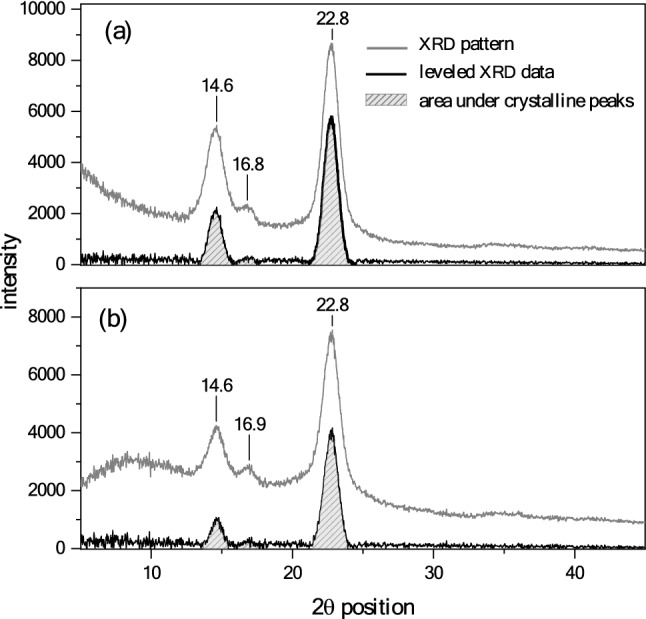
XRD spectra used for crystallinity index calculation (origin Pro software): (**a**) BC and (**b**) BC-based CH.

A comparison between the microfibril network of BC and plant cellulose (PC) indicates that the quality and extent of hydrogen bonding formation are more under the influence of molecular pathways in BC synthesis than in PC^3^. Figure 2 shows SEM images of the surfaces of BC and BC-based CH. XRD pattern of the BC shown in Fig. 3 presents three peaks at 14.6°, 16.8°, and 22.8°. BC-based CH exhibits lower-intensity peaks in the XRD pattern that are comparable to those of BC, suggesting a decrease in the crystallinity of the composite hydrogel. The calculated values for the crystallinities of the BC and BC-based CH were 63.6% and 57.8%, respectively, using Origin Pro software.

**Figure 4:**
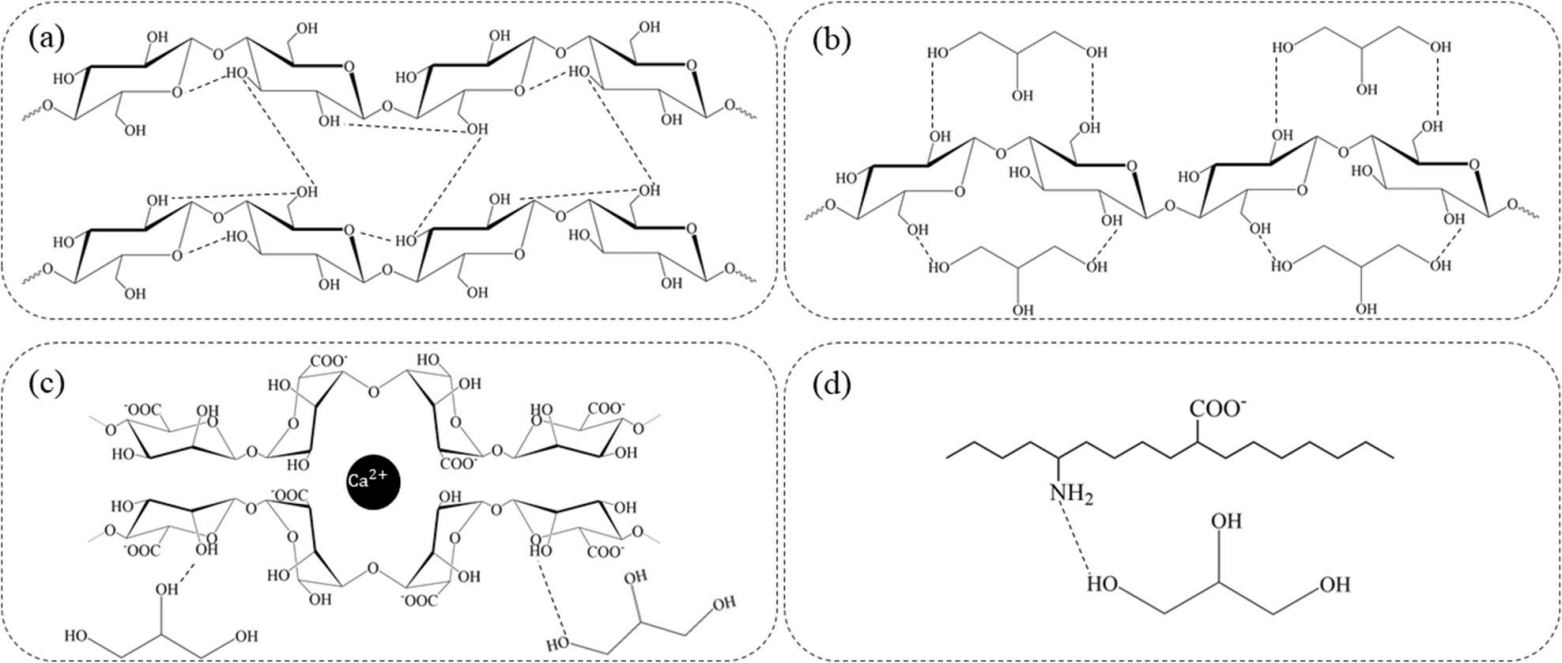
Polymers were adjustable to change structurally using glycerol (CH_2_OH–CHOH–CH_2_OH). Hydrogen bonding formation (inter/intra-linkages) is shown schematically for (**a**) bacterial cellulose, (**b**) bacterial cellulose plus glycerol, (**c**) calcium alginate plus glycerol, and (**d**) gelatin plus glycerol.

As has mentioned above, the result of closely packed cellulose microfibrils and their togetherness is the formation of the ribbons structures stacked on each other and being placed outside the bacterial cell, and the involvement of hydrogen bonding is structural behavior attributed to the rigidity of BC (Fig. 4a). Moreover, calcium alginate chain rigidity has been found to be under the influence of the monomer composition of the alginate, and the increase of chain flexibility was due to mannuronic monomer^26^. Dynamic behavior of polymer chains in terms of motions and movements (micro/nano-scale dimensions) and self-entanglement (such as cellulose in the native state) could be reduced in a mixture of polymers in the presence of glycerol during experimental work such as BC-based CH preparation. Thus, the chains would be less involved in entanglement conditions, and the chance of bound formation between flexible polymer chains increases (Fig. 4b–d)^27^. The positive effect of glycerol has also been reported for the BC membrane formulated to release glycolic acid for the treatment of aging diseases^28^. The favorable role of glycerol in BC/glycolic acid (GA)/glycerol (GL) membrane has been discussed in terms of the extent of compactness of the membrane, which was found to be less compact compared with BC and BC/GA membrane, but the structural porosity of the BC/GA/GL membrane was high^28^. The findings in the present study are in agreement with the aforementioned study (the average pore diameter of BC and BC-based CH based on BET analysis were measured as 8.5 nm and 14.6 nm, respectively).

**Figure 5:**
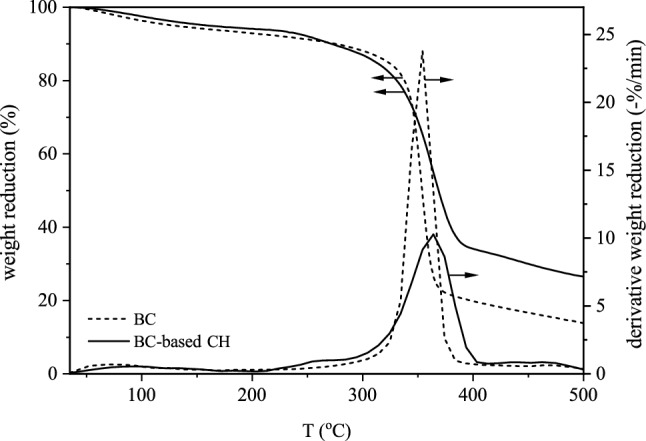
Thermal stability of BC and BC-based CH shown as TGA and first derivative curves.

The thermal behavior of BC and BC-based CH shown in Fig. 5 indicate that the extent of weight decrease in the heating process was close to each other, although the amount of weight loss at 390 °C was 12.7% higher in BC compared with BC-based CH. Weight loss of the pure BC membrane actually could occur at two stages: at 100 °C corresponded to water dehydration (physically absorbed or hydrogen-bonded to BC about 3.5%) and at about 330 °C to 390 °C corresponded to the amorphous region being interrupted (Fig. 5)^29^. The rate of change of the mass of the compound with respect to temperature is also given as a derivative curve in Fig. 5. The derivative thermogravimetric curve might be used to determine the temperature at which the greatest weight loss happens.

### Swelling behavior and MB loading experiments

**Figure 6:**
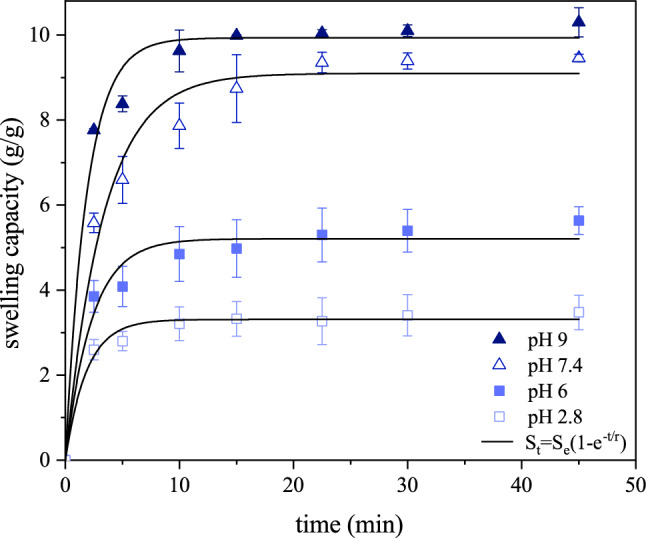
Plot of swelling capacity vs time. With use of Eq. (2), swelling rate (${\text{S}}_{\text{t}}$) of the BC-based CH was obtained for the test pHs.

Large volumes of water may be incorporated into the three-dimensional network of polymeric hydrogels without compromising their structural integrity. Drug loading and release tests, such as drug dissolution, diffusion, and transport processes, are made easier by the volume expansion of polymers or the swelling nature of hydrogels^30^. Effect of swelling media on the swelling capacity was examined considering a broad range of PH (pH 2.8–pH 9). Results of the study on the effect of pH on cellulose/whey hydrogel showed that maximum swelling capacity was when the hydrogel was soaked in distilled water at ph 7.2 (1115%) while significant decreases in swelling capacity were observed when the hydrogel was placed in an acidic medium at pH 2.5^31^. In the present study, by preparing swelling media with different pHs, the effect of pH on the swelling capacity of BC-based CH was studied. The maximum swelling capacity of the BC-based CH was at pH 9, while this capacity of the hydrogel was reduced to a minimum level at pH 2.8 (PBS as swelling media) (Table 1). The swelling capacity vs time curve presented in Fig. 6 was used to determine the swelling rate. BC-based CH, as a multi-component system, responds differently in gaining fluids from its surroundings at different pHs.

**Table 1:** The values of the equilibrium swelling (${\text{S}}_{\text{e}}$), the rate parameter (r), and the coefficient of determination (${\text{R}}^{2}$) obtained at different pHs for BC-based CH.

pH	${\text{S}}_{\text{e}}$(g/g)	r (min)	${\text{R}}^{2}$
2.8	3.31	1.86	0.98
6	5.21	2.31	0.97
7.4	9.10	3.35	0.98
9	9.93	1.89	0.99

Anionic/cationic side groups associated with these polymers depending on the ${\text{p}}{\text{K}}_{\text{a}}\text{/p}{\text{K}}_{\text{b}}$ values relative to the medium’s pH are heavily involved in the swelling process^32^. Electrostatic repulsion between the polymer chains carrying the same charges causes the gel to expand and unfold the structure, and this behavior at the microscale makes some spaces become available for receiving fluid. Further, in using calcium chloride and in cross-linking process with sodium alginate, the break-point of the egg-box of this polymer also provides spaces for calcium ions in replacement with sodium ions. Natural polymer entanglement limits the polymer’s flexibility and by introducing glycerol (sugar alcohol) to the system and the newly formed hydrogen bonds can disrupt the original bonds and this decreases chain tightness while the treated chains sense flexibility and the structure thus is less susceptible to rigidity. This behavior demonstrates how polymer properties may be altered in response to the environment. For instance, focusing on controllable drug delivery systems, stimuli-responsive nanocomposite hydrogel (NCH) has been developed by incorporating a particular form of nanosized material (such as ceramic nanoparticles, carbon-based nanoparticles, etc.). The importance of interactions between the nanosized reservoir, hydrogel matrix, and the loaded drug should be recognized both qualitatively and quantitatively^33^. The structural behavior of these so-called smart hydrogels is under the influence of environmental factors, and it can be changed to show, for instance, higher hydrophilicity, different swelling ability, etc.^34^. Thus, smart hydrogels respond adequately to pH changes, and the hydrogels are able to swell up to a desired level and also able to receive a larger amount of drug.

The chain entanglement concept is applicable to gelatin which is a denatured product of collagen. Gelatin is a mixture of amino acids in the form of polypeptides (short/long chains of peptides) and interactions between –${\text{CO}}{\text{O}}^{-}$ and –${\text{N}}{\text{H}}_{2}$ groups of gelatin chains appear to positively affect the mechanical strength/thermal stability of the formed gelatin hydrogel^35^. The results of that investigation demonstrated the significance of removing the divalent ions linked to the gelatin, and it was shown that interactions between liberated carboxylate groups and amine groups were the preferred links. To improve the mechanical and thermal stability of gelatin-based gels without focusing on the ions removal concept, BC addition to the system was also practiced using glutaraldehyde as cross-linking agent^5^. Long term structural integrity of the gelatin-based gels being prepared for encapsulating *Kluyveromyces lactis* was found to be related to using glutaraldehyde as cross-linking agent^36^.

In a research on the BC-gelatin combination in the form of sponge, Shan et al. observed that the swelling property of the generated sponge was much greater than the sample created without gelatin (3000% vs 1600%)^37^. In cases where the composite was intended for use in anti-aging skin care treatments and glycerol was included in that system, the release of the glycolic acid was delayed; it was also noted that BC/glycolic acid had an enhanced swelling capacity^28^. In vitro study further showed BC/glycolic acid/glycerol composite membrane could effectively stimulate endogenous synthesis in NIH3T3 cells (gene expression of collagen in the cells), i.e., long-term adhesion of the membrane to the cell, spreading, and enhancement of the cell division^28^. Additional research in this field focused on a transdermal medication delivery system, in which ionically modified BC when added to gelatin, might offer a lasting system for gelatin matrix, improving healing activity when using a manufactured patch^38^.

Zeta potential values of BC and BC-based CH were −2.2 and −44.56 mV, respectively, showing that BC tendency for coagulation/flocculation could be considerable. However, the suspension of BC-based CH was highly stable and acceptance of the MB as the cationic model drug was favorable.

**Figure 7:**
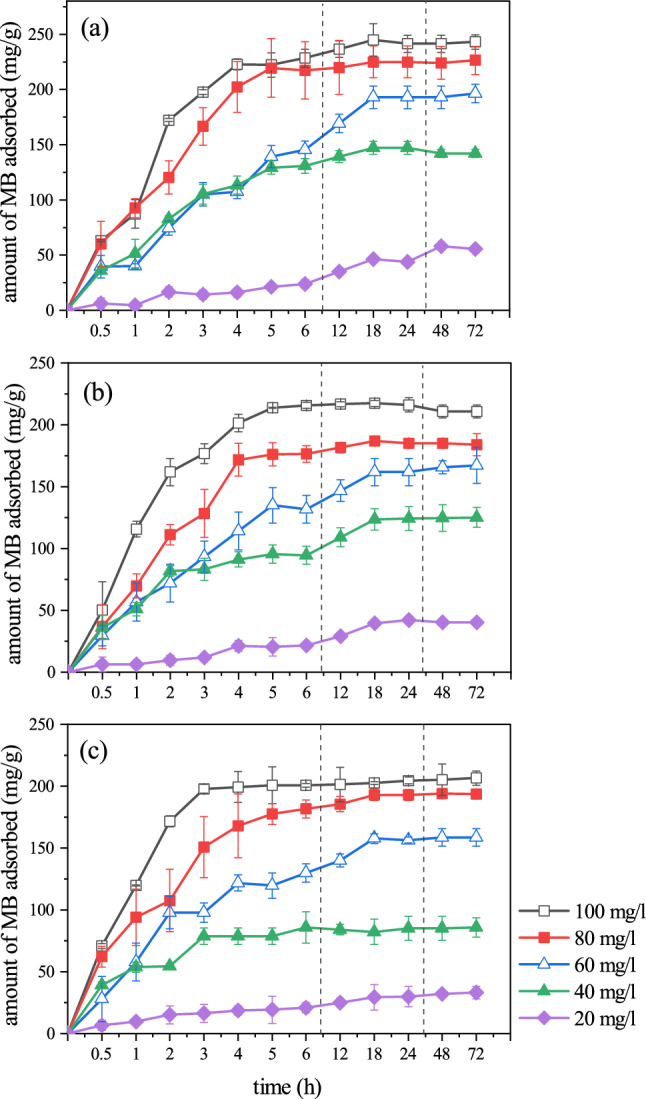
Effect of contact time for the adsorption of MB by the BC-based CH at different temperatures: (**a**) 27 °C, (**b**) 37 °C, and (**c**) 47 °C.

Therefore, efforts in the MB loading experiment were focused on describing the outcomes in terms of drug diffusion and dissolution within the polymeric network. At three different temperatures, the quantity of MB adsorbed on BC-based CH and the impact of contact duration have been determined (Fig. 7). Mixing the liquids containing MB drug/polymeric network and the disappearance of its concentration gradient explains how it is possible to have a uniform rate of drug dispersion. Further note is on the MB drug transfer via a penetrative liquid layer around BC-based CH which acts as an interface between two entities showing how drug transfer rate could determine drug dissolution rate into the polymeric network.

### Adsorption behavior of BC-based CH

#### Adsorption isotherm

Gas–solid adsorption, originally described by Langmuir as an interface phenomenon, is an energetic process and the equation is of importance in many other applications such as drug adsorption as it relates to drug delivery systems. The linearized forms of Langmuir and Freundlich equations were used in the present study:

25 $$\frac{{\text{C}}_{\text{e}}}{{\text{Q}}_{\text{e}}}{=}\frac{1}{{\text{K}}_{\text{L}}.{\text{Q}}_{\text{m}}}\text{+}\frac{{\text{C}}_{\text{e}}}{{\text{Q}}_{\text{m}}}$$

26 $${\text{ln}}{\text{ Q}}_{\text{e}}\text{ } = \text{ ln}{\text{K}}_{\text{F }}+ \text{ } \frac{1}{{\text{n}}}{\text{ln}}{\text{C}}_{\text{e}}$$

The relevant plots are presented in the Supplementary Information and values for Langmuir and Freundlich constants are given in Table 2. Langmuir equation developed based on the concept of monolayer adsorption capacity of the adsorbent was adequately used to find the maximum adsorption capacity (${\text{Q}}_{\text{m}}$) of the BC-based CH as the adsorbent for the MB in the aqueous solution as the adsorbate molecule. The calculated ${\text{Q}}_{\text{m}}$ value was the highest at ${47} \, \circ \text{C}$ (366 mg/g), while the affinity of the MB for the adsorbent (i.e., Langmuir constant) was higher at 27 °C compared to two other test temperatures: ${\text{K}}_{{\text{L}}_{\text{47}}}\circ {\rm C} { < }{\text{K}}_{{\text{L}}_{\text{37}}} \circ {\rm C} { < }{\text{K}}_{{\text{L}}_{\text{27}}} \circ {\rm C}$. Values of ${\text{Q}}_{\text{m}}$ decreased with increasing adsorption temperature from 27 to 37 °C, while the ${\text{Q}}_{\text{m}}$ increased when the temperature increased further to 47 °C (Table 2). In the Langmuir model, emphasis is on having an energetically equivalent site distributed uniformly on the adsorbent surface as a monolayer and available for making chemical bonds with the adsorbate molecules. These bonds in chemisorption and physisorption are both temperature-dependent. BC-based CH as a multi-component adsorbent contained gelatin and gelatin potentiality of gelation at a higher temperature than 27 °C, negatively affected the adsorbent where the increased movements of the dissolved MB occupied the surface leading to MB desorption. Equality of adsorption rate with the desorption rate is a central point in defining the Langmuir equation and the potentiality of keeping this condition at 37 °C was considerably decreased and the MB desorption was favored. Some structural difficulties in the polymeric network are the result of the MB loss and the appearance of empty sites which may become active in a kind of exchange process and as exchangeable sites could participate in the MB adsorption and the ${\text{Q}}_{\text{m}}$ increased at 47 °C because of the involvement of these newly formed sites in the MB sorption. The ability of BC-based CH adsorbent to keep adsorbed molecules, however, was low and the ${\text{K}}_{\text{L}}$ value was the lowest at 47 °C (Table 2). The results of the Langmuir model might be better understood if varied dosages of the polymeric adsorbent were examined. According to Langmuir concept, ${\text{R}}_{\text{L}}$ value is an appropriator constant showing whether a particular adsorption process is suitable or not. ${\text{R}}_{\text{L}}$ values presented in Table 2 indicate that the MB adsorption onto BC-based CH was carried out favorably. Results of using the Freundlich model are also presented in Table 2. Physisorption is the basis for this empirical equation where findings in terms of multilayer adsorption in addition to monolayer are explained. The quality of the heterogeneous adsorbent surface is defined by 1/n value as the intensity factor and ${\text{K}}_{\text{F}}$ as the adsorption capacity. The value of 1/n was higher at 47 °C and expectedly the adsorption capacity was lower at that temperature. It is important to monitor how the adsorbent responds to the adsorbate in terms of its nature. Physical adsorption is explained by van der Waals forces, which are characterized in terms of the distances between the involved atoms/molecules and these forces grow less at high temperatures. The value of the ${\text{K}}_{\text{F}}$ was lower at 47 °C compared to other test temperatures (more than 75% decrease in the adsorption capacity) (Table 2).

**Table 2:** Langmuir and Freundlich constants of MB adsorption on BC-based CH at different temperatures.

Temperature (°C)	Langmuir				Freundlich		
	${\text{Q}}_{\text{m}}$	${\text{K}}_{\text{L}}$	${\text{R}}_{\text{L}}$	${\text{R}}^{2}$	$\text{1/n}$	${\text{K}}_{\text{F}}$	${\text{R}}^{2}$
27	267.37	0.277	0.034	0.99	0.25	97.09	0.95
37	244.71	0.095	0.082	0.99	0.29	64.40	0.98
47	367.68	0.022	0.299	0.97	0.62	17.08	0.96

${\text{Q}}_{\text{m}}$: mg/g, ${\text{K}}_{\text{L}}$: l/mg, ${\text{K}}_{\text{F}}$: mg/g, ${\text{R}}^{2}$: the correlation coefficient values being expressed in model fitting process.

### Thermodynamic quantities

**Figure 8:**
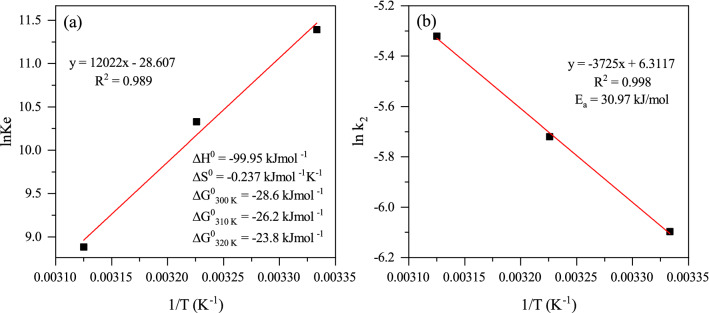
(**a**) The van’t Hoff thermodynamic plot and (**b**) Arrhenius equation used to plot ${{\text{ln}}{\text{k}}}_{2}$ vs 1/T for the adsorption of MB onto BC-based CH.

Placing Langmuir constant (${\text{K}}_{\text{L}}$) instead of ${\text{K}}_{\text{eq}}$ is problematic and the issue was discussed by several works reported in the literature and the approach taken by Ghosal et al. was considered in the present study^39^: Langmuir constant (L/mg) multiplied by the molar weight of MB (mg/mol) as the adsorbate was placed instead of the equilibrium constant as mentioned in the Eq. (8). Thermodynamic quantities calculated from the van’t Hoff equation are presented in Fig. 8a. A negative value of the enthalpy change (−99.953 ${\text{kJ}} \, {\text{mo}}{\text{l}}^{-{1}}$) indicates that the nature of the adsorption was exothermic, i.e., heat is released during the process. The entropy change is not much less than zero (−0.237 ${\text{k}}{\text{J}} \, {\text{mo}}{\text{l}}^{-{1}} {\text{K}}^{-{1}}$) and indirectly indicates that the MB sorption onto the BC-based CH surface (at the solid–liquid interface) is in the boundary between two zones of a more disordered interface to a less disordered interface. Further note is on the spontaneity of the adsorption process, which is under the influence of temperature (Fig. 8a): $\Delta {\text{G}}_{\text{47}} \circ C > \Delta {\text{G}}_{\text{37}} \circ C > \Delta {\text{G}}_{\text{27}} \circ C$.

### Process activation energy

The obtained adsorption rate constants were used for the calculation of activation energy (${\text{E}}_{\text{a}}\text{)}$ according to the Arrhenius equation (see the Supplementary Information):

27 $$k=A\mathrm{exp}(-{E}_{a}/RT)$$

The calculated activation energy at 30.97 kJ/mol indicates that the rate of MB adsorption on the BC-based CH adsorbent was relatively fast^40^. Freundlich adsorption, in which both monolayer (chemisorption)/multilayer (physisorption) coverage are involved, appears to be in a better position compared to the Langmuir equation for describing the results (Fig. 8b).

### Release kinetics and relevant results of the TOPSIS algorithm

**Figure 9:**
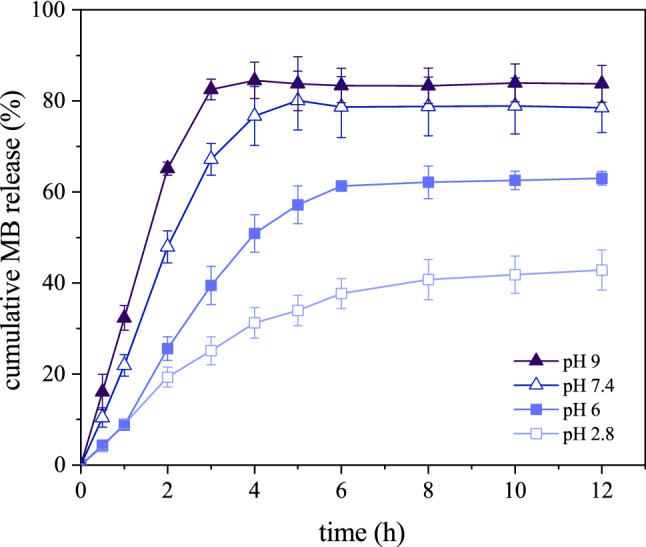
Cumulative MB release from the BC-based CH at different pHs.

The profile of the cumulative release of MB from the BC-based CH samples at four test pHs shown in Fig. 9 indicates that by raising the pH from 2.8 to pH 9, the ultimate cumulative MB release (CR) from the test composites increased (${\text{C}}{\text{R}}_{\text{pH 2.8}} \, {=} \, \text{42.8}\%$, ${\text{C}}{\text{R}}_{\text{pH 6}} \, {=} \, \text{63} \%$, ${\text{C}}{\text{R}}_{\text{pH 7.4}} \, {=} \, \text{80}\%$, and ${\text{C}}{\text{R}}_{\text{pH 9}} \, {=} \, \text{84.5}\%$). This trend follows favorably the results obtained from swelling behavior as shown in Fig. 6.

An acceptable approach in predicting drug release from a network is to use appropriate mathematical models. The data obtained for MB release from the BC-based CH were fitted into five different models (Eqs. 9–13). In this way, the kinetic behavior of the MB release mechanism was explained.

Tha data obtained in the release experiments were also processed with use of the Hopfenberg model, which is developed based on the surface erosion of drug carriers (Table 3). Natural polymers are utilized in synthesizing the BC-based CHs, and in vivo study on MB release from these hydrogels may give some useful results.

**Table 3:** Specifications of the mathematical modeling for MB release from BC-based CH.

Model type	Model parameters and model selection criteria^a^	pH			
		2.8	6	7.4	9
$\frac{{\mathrm{M}}_{\mathrm{t}}}{{\mathrm{M}}_{\infty }}={\mathrm{kt}}^{\mathrm{n}}$ (Korsmeyer–Peppas)	K	0.20	0.16	0.27	0.37
	N	1.05	1.26	1.06	0.94
	${\text{R}}_{\text{l}}^{2}$	0.992	0.992	0.998	0.993
	${\text{R}}_{\text{nl}}^{2}$	0.986	0.994	0.996	0.986
	RMSE	0.0231	0.0826	0.1435	0.0849
	χ^2^	0.007812	0.004501	0.003283	0.010215
	RSS	0.00214	0.00109	0.00134	0.00545
	AIC	−20.588	−23.286	−22.460	−16.849
$\frac{{\mathrm{M}}_{\mathrm{t}}}{{\mathrm{M}}_{\infty }}={\mathrm{kt}}^\frac{1}{2}$ (Higuchi)	K	0.46	0.56	0.67	0.79
	${\text{R}}_{\text{l}}^{2}$	0.994	0.988	0.992	0.994
	${\text{R}}_{\text{nl}}^{2}$	0.819	0.714	0.800	0.853
	RMSE	0.0165	0.1183	0.1491	0.1207
	χ^2^	0.102626	0.195719	0.157618	0.125496
	RSS	0.02731	0.05602	0.06123	0.05667
	AIC	−12.402	−9.5282	−9.1725	−9.4820
$\frac{{M}_{t}}{{M}_{\infty }}=1-{\left[1-\frac{{k}_{0}^{{\prime}}t}{{C}_{0}a}\right]}^{m}$ (Hopfenberg)	${\text{k}}_{0}^{\prime}{ (}{\text{mg}}/{\text{cm}}^{2}\text{h)}$	0.082	0.094	0.100	1.131
	${\text{R}}_{\text{l}}^{2}$	0.983	0.994	0.996	0.979
	${\text{R}}_{\text{nl}}^{2}$	0.983	0.973	0.996	0.969
	RMSE	0.0183	0.1237	0.2025	0.2222
	χ^2^	0.006384	0.029196	0.003202	0.017792
	RSS	0.0026	0.00528	0.00134	0.01181
	AIC	−21.809	−18.975	−24.460	−15.755
$\frac{{\mathrm{M}}_{\mathrm{t}}}{{\mathrm{M}}_{\infty }}=\mathrm{kt}$ (zero-order)	K	0.20	0.23	0.29	0.32
	${\text{R}}_{\text{l}}^{2}$	0.983	0.994	0.996	0.979
	${\text{R}}_{\text{nl}}^{2}$	0.983	0.973	0.996	0.969
	RMSE	0.0183	0.1237	0.2025	0.2222
	χ^2^	0.006384	0.029196	0.003202	0.017792
	RSS	0.0026	0.00528	0.00134	0.01181
	AIC	−21.809	−18.975	−24.460	−15.755
$\frac{{\mathrm{M}}_{\mathrm{t}}}{{\mathrm{M}}_{\infty }}=1-{\mathrm{e}}^{-\mathrm{kt}}$ (first-order)	K	0.71	0.88	0.72	0.64
	${\text{R}}_{\text{l}}^{2}$	0.899	0.953	0.928	0.904
	${\text{R}}_{\text{nl}}^{2}$	0.978	0.910	0.926	0.915
	RMSE	0.0369	0.119	0.2364	0.2102
	χ^2^	0.017567	0.072554	0.059653	0.064871
	RSS	0.00337	0.01769	0.02277	0.03285
	AIC	−20.771	−14.139	−13.129	−11.663

^a^${\text{R}}_{\text{l}}^{2}$: coefficient of determination for linear regression, ${\text{R}}_{\text{nl}}^{2}$: coefficient of determination for non-linear regression, *RSS* residual sum of squares.

Further note was to examine the extent of diffusion mechanism application, and this was based on the interpretation of the release exponent ‘n’ calculated from the fitting data into the Korsmeyer-Peppas model^15,41^: n < 0.5—quasi-Fickian diffusion mechanism, n = 0.5—diffusion mechanism, 0.5 < n < 1—non-Fickian diffusion, n = 1—case II transport/ zero-order release, and n > 1—super case II transport.

To describe the drug release kinetic from a polymeric network, drug dissolution, diffusion, and transport mechanisms are all involved, while considerations of the network absorption capacity, swelling behavior, erosion, and even degradation are necessary. Focusing on the value of the release exponent ‘n’ in Korsmeyer–Peppas model does not provide enough information on the release.

It was fairly reasonable to consider non-linear along with linear fitting to treat different kinetic models used for the MB release in the present study (Table 3).

**Figure 10:**
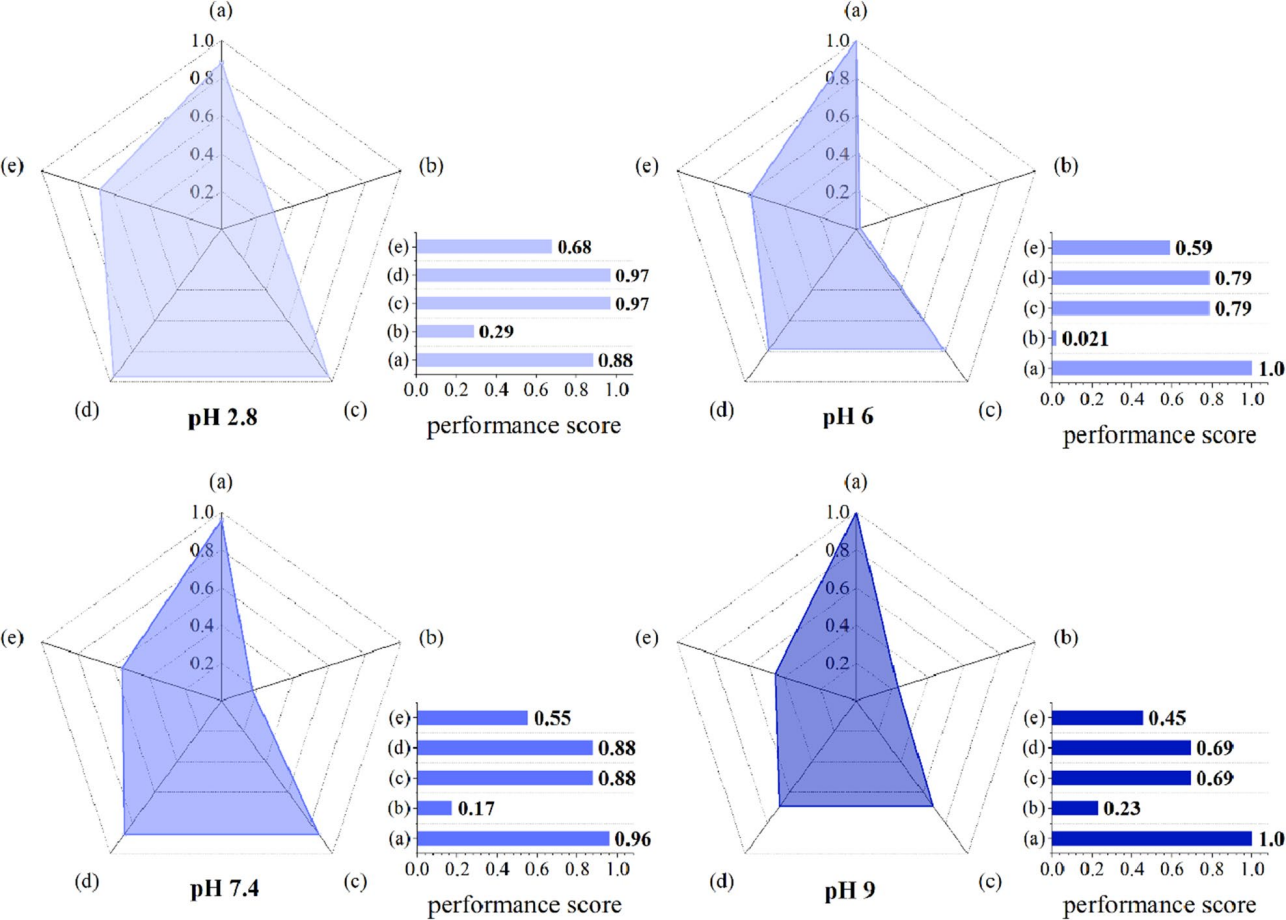
Plots of performance scores (${\text{p}}_{\text{i}}$) which were calculated based on the TOPSIS algorithm used for release kinetic model assessment at different pHs: (**a**) Korsmeyer–Peppas, (**b**) Higuchi, (**c**) Hopfenberg, (**d**) zero-order equation, and (**e**) first-order equation.

This is a reasonable expectation to see error being generated in any data collection process especially when one focuses on a research study conducted based on the experimental work to obtain results of the relationship between several variables. ${\text{R}}^{2}$, χ^2^, RMSE, and AIC as error criteria were used in the present study to estimate error for the model prediction process (non-linear treatment of data), where each model was used to quantify BC-based CH performance in releasing MB in release media at different pHs. The rational basis for handling such data appropriately is to use the TOPSIS method, which by describing a simple mathematical expression, the algorithm can provide a logical explanation for the selected variable^42^. Knowledge gained in the process is based on defining an ideal solution, and the extent of the closeness of the test variable to the ideal solution shows a preferred position for one variable over other test variables. High flexibility of TOSIS concept helps one to efficiently participate in the decision-making process. For instance, the TOPSIS results shown in Fig. 10 indicate that the value of ${\text{p}}_{\text{i}}$ as the model performance quality (Eq. 24) was higher than 0.88 for Korsmeyer-Peppas model at all pHs. The Higuchi model had lower ${\text{p}}_{\text{i}}$ value (< 0.29) at all test pHs. By increasing pH of the release medium from 2.8 to pH 9, the ${\text{p}}_{\text{i}}$ value for the first-order kinetic model showed a decreasing trend from 0.68 to 0.45.

By changing the weight of each criterion in the criteria set used to evaluate the model performance, the sensitivities of release models (alternatives) at different pHs will be changed, and this appears to be influential on TOPSIS results.

## Conclusions

BC-based CHs formed resulted from physically cross-linked BC, Alg, and Gl, where with the use of glycerol, the polymers’ chains were flexible and adjustable to change structurally. With the use of instrumental analyses, the hydrogel was characterized structurally. The behavioral functionalities of developed hydrogels due to swelling, adsorption capacity, and thermodynamic properties were explainable quantitatively. MB release media were prepared at different pHs, and with the use of five models, the release kinetics were characterized. The data fitting process was performed linearly and non-linearly, where the TOPSIS algorithm was found to be beneficial in making a decision on model predictability as related to pH. The TOPSIS algorithm usage appears to be supportive for result interpretation, especially when data were treated non-linearly, using different error criteria such as ${\text{R}}^{2}$, χ^2^, RMSE, etc. According to the obtained results, the prepared composite hydrogel could be an appropriate candidate for drug delivery applications.

## Supplementary Information

Supplementary Information.

## Data Availability

All data generated and/or analyzed during the current study are included in the submitted manuscript and its Supplementary Information files. Further raw data is also available in the figshare repository, https://doi.org/10.6084/m9.figshare.21725303.
