# Supplementary material for: Application of TOPSIS algorithm in describing bacterial cellulose-based composite hydrogel performance in incorporating methylene blue as a model drug
Source: Sci Rep. 2023 Feb 16;13:2755. doi: 10.1038/s41598-023-29865-6 (PMC9935555; doi:10.1038/s41598-023-29865-6)
Supplement: Supplementary file 1 — Supplementary Information. [file 41598_2023_29865_MOESM1_ESM.docx]

**Application of TOPSIS algorithm in describing bacterial cellulose-based composite hydrogel performance in incorporating methylene blue as a model drug**

Touraj Amrabadi^a^, Elham Jalilnejad^b*^, Seyed Mohammad Amin Ojagh^a,c^, Farzaneh Vahabzadeh^a^

^a^ Department of Chemical Engineering, Amirkabir University of Technology (Tehran Polytechnic), Tehran, Iran.

^b^ Department of Chemical Engineering, Urmia University of Technology, Urmia, West Azerbaijan, Iran

^c^ Department of Chemistry, McGill University, Montreal, Quebec, Canada.

^*^ Corresponding author.

*E-mail address:* e.jalilnejad@uut.ac.ir (E. Jalilnejad)

**Supplementary Information: models used for describing adsorption isotherm and its kinetics**

# Adsorption isotherm models

**Fig. S.1.** linearized forms of Langmuir and Freundlich adsorption isotherm where the plot shows experimental data (■) and the model fitted data (––). The data was obtained at three different temperatures: (a) 27°C, (b) 37°C, and (c) 47°C.

# Adsorption kinetics and rate constant determination

In the adsorption process quantification, MB at different concentrations (20-100 mg/l) were used in the adsorption experiments and in each case, the amount of MB at the equilibrium position ($\text{q}_{\text{e}}$) and at time t during adsorption ($\text{q}_{\text{t}}$) were determinable. Two commonly used kinetic models in the linearized forms namely pseudo-first-order (PFO) and pseudo-second-order (PSO) equations were used:

| $\ln\left( q_{e}-q_{t} \right)=-k_{1}t+\ln q_{e}$ | (S.1) |
| --- | --- |
| $\frac{t}{q_{t}}=\frac{1}{k_{2}q_{e}^{2}}+\frac{t}{q_{e}}$ | (S.2) |

And plotting $\ln\text{(q}_{\text{e}}\text{-}\text{q}_{\text{t}}\text{)}$ as a function of t yields the intercept and slope terms as $\text{q}_{\text{e}}$ and $\text{k}_{\text{1}}$, respectively. While plotting $\text{t/}\text{q}_{\text{t}}$ vs. time yields $\text{k}_{\text{2}}$ and $\text{q}_{\text{e}}$ as the intercept and slope, respectively (**Fig. S.2**) ^1^.

**Fig. S.2** Kinetic models were used in the present study (PFO and PSO) for the calculation of the relevant rate constants. MB at 100 mg/l was used and the experiments were performed at (a) 27°C, (b) 37°C, and (c) 47°C.

Steadily increase in the rate constant from 27°C to 47°C was more clearly sensed in the PSO model compared to that in the PFO model, and the findings in the PSO were used for plotting the Arrhenius equation ($\text{k=A}\exp{\text{(-E}_{\text{a}}}/\text{RT}\text{)}$) where A is the pre-exponential factor and $\text{E}_{\text{a}}$ is the activation energy (**Table S.1**).

**Table S.1.** Kinetic parameters for the adsorption of MB onto BC-based CH obtained according to the PFO and the PSO models

| kinetic model | rate constant |  | T (K) |  |
| --- | --- | --- | --- | --- |
|  |  | 300 | 310 | 320 |
| PFO | $\text{k}_{1}$ ($\text{h}^{\text{-1}}$) | 0.594 | 0.625 | 0.993 |
| PSO | $\text{k}_{\text{2}}$ (g/mgh) | 0.00225 | 0.00328 | 0.00489 |

**References**

1. William Kajjumba, G., Emik, S., Öngen, A., Kurtulus Özcan, H. & Aydın, S. Modelling of Adsorption Kinetic Processes—Errors, Theory and Application. in *Advanced Sorption Process Applications* (IntechOpen, 2019). doi:10.5772/intechopen.80495.
